# Efficacy and Safety of iLet Bionic Pancreas in Patients With Type 1 Diabetes Mellitus: A Systematic Review and Meta Analysis

**DOI:** 10.1002/edm2.70127

**Published:** 2025-10-28

**Authors:** Sunny Kumar, F. N. U. Aakash, Nisha Kumari, Chandar Kanta Lohana, Alina Abbas, F. N. U. Gyaneshwari, Raveena Kumari, F. N. U. Eman, Reena Bai, Saifullah Syed, Mahveer Maheshwari, Rahul Rai, Faiqa Iqbal, Mohammad Jawwad, Hira Riaz

**Affiliations:** ^1^ Wright Center for Graduate Medical Education Scranton Pennsylvania USA; ^2^ Florida State University Tallahassee Florida USA; ^3^ Liaquat National and Medical College Karachi Pakistan; ^4^ King Edward Medical University Lahore Pakistan; ^5^ United Medical and Dental College Karachi Pakistan; ^6^ Royal College of Surgeons Dublin Ireland; ^7^ Shaheed Mohtarma Benazir Bhutto Medical College Karachi Pakistan; ^8^ Dow University of Health and Sciences Karachi Pakistan; ^9^ Kabul University of Medical Sciences Abu Ali Ibn Sina Kabul Afghanistan

**Keywords:** continuous glucose monitoring, endocrine disorder, iLet bionic pancreas, insulin, type 1 diabetes

## Abstract

**Background:**

This systematic review and meta‐analysis evaluated the efficacy and safety of the iLet bionic pancreas (iLet BP), a novel automated insulin delivery (AID) system, in managing type 1 diabetes. Unlike conventional AID systems, which require user input for insulin dosing, the iLet BP autonomously determines insulin delivery based solely on body weight. The study synthesized data from five randomized controlled trials (RCTs), comprising a total of 1130 patients, comparing iLet BP with standard care (SC).

**Outcomes Assessed:**

Primary outcomes included changes in HbA1c, mean glucose levels, and time in target glucose range (70–180 mg/dL), measured via continuous glucose monitoring (CGM). Secondary outcomes assessed adverse events and hypoglycaemia.

**Key Findings:**

Results demonstrated that the iLet BP significantly improved glycaemic control. The pooled analysis showed a standardised mean difference (SMD) in HbA1c of −0.50 [−0.63, −0.38] and in mean glucose levels of −0.36 [−0.50, −0.21] favouring iLet BP. Time in target glucose range was significantly higher with iLet BP (SMD: 0.58 [0.43, 0.73]). However, the odds of adverse events were notably higher in the iLet BP group (OR: 15.48 [8.07, 29.70]), while the risk of hypoglycaemia (OR: 2.22 [0.83, 5.94]) was not statistically significant.

**Conclusion:**

In conclusion, the iLet BP shows strong potential in improving glycaemic outcomes in patients with type 1 diabetes. However, concerns remain regarding its safety profile, particularly related to adverse events. Further large‐scale, high‐quality studies are needed to confirm its effectiveness and ensure broader clinical applicability.

## Graphical abstract

Figure illustrating the summary of efficacy and safety of iLet bionic pancreas in patients with type 1 diabetes mellitus.

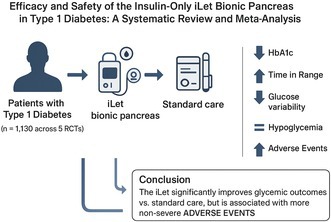

**Abbreviations**
AID: automated insulin delivery
CGM: continuous glucose monitoring
DKA: diabetic ketoacidosis
HbA1C: glycated haemoglobin
HCL: hybrid closed loop
iLet BP: iLet bionic pancreas
MDI: multiple daily injections
OR: odds ratio
RCT: randomised controlled trial
SC: standard care
SH: severe hypoglycaemia
SMD: standard mean difference
TIR: Time in range 54–10 mg/dL

## 1 Introduction

Type 1 diabetes is a result of autoimmune destruction of insulin‐producing β‐cells in the pancreas, with genetic and multiple environmental factors acting as precipitators of the disease. The high mortality associated with the prognosis of type 1 diabetes and the increasing incidence of type 1 diabetes emphasize the importance of therapeutic strategies to prevent this chronic disorder [1].

Automated insulin delivery systems have been developed to manage type 1 diabetes. The systems that have become commercially available over the past 5 years (Medtronic MinimedTM 670G and 780G [2], Tandem t: slim X2 with Control‐IQ Technology, and Insulet Omnipod 57) are referred to as hybrid closed loop (HCL) systems. They partially automate insulin delivery but still require insulin titration and dosing decisions on the part of the health care provider and user [3].

Modern innovations in diabetes technology have transformed the management of type 1 diabetes. Among these, the iLet bionic pancreas (Beta Bionics Inc.) represents a next‐generation advancement following the development of HCL systems. The iLet bionic pancreas (iLet BP) is a purpose‐built, fully integrated device that communicates directly with a continuous glucose monitor (CGM) and incorporates autonomous, lifelong‐learning dosing algorithms initialized only with the patient's body weight [4]. Unlike earlier systems, it does not require information about prior insulin dosing [5]. All insulin titration—including basal, correction and meal‐announcement doses—is determined solely by the iLet algorithms, which continually adapt to the individual's insulin needs and cannot be modified by the user or healthcare provider [5].

CGMs themselves play a central role in modern diabetes care. By continuously tracking blood glucose levels in real time through a subcutaneous sensor, they provide trend data, alert patients to hypo‐ and hyperglycaemia, and reduce the burden of frequent finger‐stick testing. Their use has been associated with improved glycaemic control and more precise insulin dosing decisions [6].

HCL systems, which partially automate insulin delivery, have demonstrated significant benefits in reducing both hyperglycaemia and hypoglycaemia in adults and children with type 1 diabetes [1]. However, hyperglycaemia remains common, with many patients spending 6–7 h per day above 180 mg/dL, underscoring the need for systems that can achieve full automation of insulin delivery [5]. Newer automated insulin delivery (AID) technologies, including the iLet, are advancing this paradigm by reducing the need for user input at both device initialization and during daily use [7].

To our knowledge, no previous meta‐analysis has compared the efficacy of the insulin‐only iLet BP with standard care in patients with type 1 diabetes. The purpose of this study is to evaluate the effectiveness of the iLet BP compared with standard care, and to assess potential risks associated with its use in individuals with type 1 diabetes.

## 2 Methods

### 2.1 Study Design and Protocol

We conducted a systematic review and meta‐analysis according to the Preferred Reporting Items for Systematic Reviews and Meta‐Analyses (PRISMA) statement [8]. The study was submitted to OSF with a registration code: https://doi.org/10.17605/OSF.IO/2VY3H.

### 2.2 Data Source and Search Strategy

We conducted a broad literature search to identify studies on iLet bionic pancreas. To assess the evidence for this purpose, a broad search for clinical trials was initiated through Pubmed, Web of Science, and Scopus databases for the trials published up to July 3, 2025, by two independent authors. The electronic search strategy included both Medical Subject Headings (MeSH) and keywords (free text words). Search terms included the keyword terms ‘iLet’, ‘Bionic pancreas’, ‘Artificial pancreas’. Any discrepancies (if present) were resolved by a third author. The study was limited to human subjects and the articles were written in English. The literature was managed, and duplicates were removed using EndNote X7 software. The reference lists and topic‐related reviews were manually checked to identify relevant papers. Further details on search strategy are given in Table 1.

**TABLE 1:** Electronic database search strategies used for identifying studies on the iLet bionic pancreas in type 1 diabetes.

Database	Search date	Search terms	Limits applied	Results retrieved
PubMed	1 March 2025	(‘Bionic Pancreas’ OR ‘iLet’ OR ‘automated insulin delivery’ OR ‘artificial pancreas’) AND (‘Type 1 Diabetes’ OR ‘T1DM’) AND (randomised OR RCT)	Humans, English	361
Web of Science	1 March 2025	ALL = ([‘Bionic Pancreas’ OR ‘iLet’] AND [‘Type 1 Diabetes’])	Article, English	256
Scopus	1 March 2025	TITLE‐ABS‐KEY (‘Bionic Pancreas’ OR ‘iLet’) AND TITLE‐ABS‐KEY (‘Type 1 Diabetes’)	Human studies, English	233
Total				850

#### 2.2.1 Study Selection, Inclusion and Exclusion Criteria

We included only randomised controlled trials (RCTs), as they provide the highest level of evidence and minimise potential bias when evaluating clinical interventions. Studies were excluded if they were case reports, reviews, letters or conference abstracts without full text. Two authors independently screened titles and abstracts, followed by full‐text review. Disagreements were resolved through discussion with a third author. All included studies represented unique trials. The PICO (Patient/Population, Intervention, Comparison, and Outcomes) framework guided the inclusion criteria for eligible original articles.

### 2.3 Population

Patients diagnosed with type 1 diabetes mellitus (T1DM) of any age or gender who require insulin therapy for glycaemic control. The inclusion of participants using conventional insulin delivery methods as a comparator was noted.

### 2.4 Intervention

The use of the iLet bionic pancreas, an autonomous insulin delivery system designed to regulate blood glucose levels with minimal manual adjustments, is outlined here. The device integrates continuous glucose monitoring (CGM) with an insulin pump to optimise glycaemic control. Unlike conventional therapy, it removes the need for carbohydrate counting, but users are still required to announce meals (e.g., typical, more or less than usual). Studies assessing insulin‐only iLet configurations will be included. The primary goal of this intervention is to improve glycaemic outcomes compared to conventional insulin therapy.

### 2.5 Comparison

The efficacy and safety of the iLet bionic pancreas was compared to standard care, which included multiple daily injections (MDI), conventional insulin pump therapy, or hybrid closed‐loop (HCL) systems, with or without continuous glucose monitoring (CGM). Standard care required patients to manually adjust insulin doses based on glucose readings, carbohydrate intake and activity levels, whereas the iLet system automated insulin delivery and reduced the burden of meal‐related calculations, though meal announcements were still required.

### 2.6 Outcomes

Primary outcomes included changes in glycated haemoglobin (HbA1c) and CGM metrics, such as time spent in different glucose ranges (e.g., hypoglycaemia, euglycaemia and hyperglycaemia). Additional primary measures will include the coefficient of variation (CV) and standard deviation (SD) of glucose levels to assess glycaemic variability. Secondary outcomes included any adverse events, events of hypoglycaemia and events of diabetic ketoacidosis.

Details of each outcome measured are as follows:

### 2.7 Primary Outcomes

#### 2.7.1 HbA1c

One of the primary glycaemic outcomes assessed was the change in glycated haemoglobin (HbA1c) from baseline to 13 weeks of follow‐up. HbA1c was measured using standard laboratory assays at study initiation (pre‐intervention) and again at the end of the 13‐week study period (post‐intervention) for both the iLet Bionic Pancreas (intervention) and standard care (control) groups. The difference between the baseline and post‐intervention HbA1c values was calculated within each iLet Bionic Pancreas and standard care group, and comparison was performed between them by plotting the standard mean difference in forest plots.

#### 2.7.2 Time in Euglycaemic Range (70–180 mg/dL)

Time in range (TIR), defined as the percentage of time that glucose values remained within the target range of 70–180 mg/dL, was a key continuous glucose monitoring (CGM)‐derived outcome used to assess glycaemic control. CGMs (Dexcom G6) provided real‐time interstitial glucose data and were worn by all participants throughout the study. CGM devices measured glucose values every 5 min, yielding up to 288 readings per day. These high‐frequency data were used to ensure stable glycaemic patterns and to cross‐validate trends associated with HbA1c changes. Participants in both groups maintained ≥ 80% CGM data capture during the 13‐week study period, ensuring reliability in comparing mean glucose levels and other CGM‐derived metrics. TIR was calculated using data obtained from CGM devices worn by participants in both the iLet BP (intervention) and standard care (control) groups. Baseline TIR was established using CGM data collected during the run‐in or preoperative phase. At the end of the 13‐week intervention period, post‐treatment TIR was again assessed using CGM data collected over the final 1–2 weeks of the study. The difference in TIR from baseline to follow‐up was calculated for each group, and comparison was performed between them by plotting standard mean difference (SMD) in forest plots.

#### 2.7.3 Time Spent in Hypoglycaemia and Hyperglycaemia

Additional glycaemic outcomes included the percentage of time spent in hypoglycaemia (glucose < 70 and < 54 mg/dL) and hyperglycaemia (glucose > 180 and > 250 mg/dL), derived from continuous glucose monitoring (CGM) data. These outcomes were calculated from CGM readings collected using the Dexcom G6 system, which captured interstitial glucose levels every 5 min, providing granular data for daily glucose excursions. The mean difference in time spent in each glucose range < 54, < 70, > 180, > 250 mg/dL from baseline to follow‐up was calculated for each group, and comparison was performed between them by plotting standard mean difference (SMD) in forest plots.

### 2.8 Secondary Outcomes

#### 2.8.1 Glucose Variability and Coefficient of Variation

Glucose variability was assessed using two CGM‐based metrics: the standard deviation (SD) of glucose levels and the coefficient of variation (CV). These measures were recorded at baseline and after 13 weeks of intervention. Standard deviation reflects absolute variability in glucose, while CV (calculated as SD divided by mean glucose) accounts for relative fluctuations, offering a normalised index of glycaemic instability. Both outcomes were compared between the iLet BP and standard care groups, and pooled using standardised mean differences (SMDs) in the meta‐analysis.

#### 2.8.2 Adverse Events

The occurrence of any adverse events (AEs) during the 13‐week study period was recorded for both intervention and control groups. Adverse events were defined as any untoward medical occurrences temporally associated with device use or diabetes management, regardless of causal relationship. Participants and caregivers reported AEs through structured clinical interviews and electronic monitoring systems, and events were adjudicated by the study investigators. Reported AEs included infusion set failures, skin irritation, gastrointestinal disturbances, and mild infections, hypoglycaemia, among others. For the purposes of meta‐analysis, adverse events were treated as a dichotomous outcome (presence or absence of any AE), and odds ratios (ORs) were calculated to compare the frequency of events between the iLet and standard care groups. The heterogeneity and relative severity of events are discussed in the Limitations section, as these factors may influence the interpretation of safety outcomes.

#### 2.8.3 Hypoglycaemic Events

Hypoglycaemic events were captured as symptomatic or clinically significant episodes, typically defined by a glucose level < 70 mg/dL accompanied by symptoms or requiring assistance. These events were reported through patient diaries, device logs (via CGM or insulin pump alerts), or investigator assessments during scheduled visits. Each participant's data were assessed for the occurrence of at least one hypoglycaemic event during the intervention period. This outcome was analysed as a binary variable (yes/no), and odds ratios were calculated to compare the incidence of hypoglycaemic events between the iLet Bionic Pancreas and standard care arms.

#### 2.8.4 Diabetic Ketoacidosis (DKA)

Diabetic ketoacidosis (DKA) events were defined using standard clinical criteria: hyperglycaemia, metabolic acidosis, and ketonemia or ketonuria. These events were diagnosed by the clinical study team and confirmed using laboratory data when available. The occurrence of at least one DKA event per participant during the 13‐week follow‐up was recorded. For analysis, DKA was treated as a dichotomous outcome (event/no event), and odds ratios were calculated to compare the risk of DKA between intervention and control groups.

### 2.9 Screening and Data Extraction

Two independent reviewers initially screened the titles and abstracts of identified records for eligibility, followed by full‐text assessment of potentially relevant studies. Data extraction was conducted independently by two authors using a standardised Excel spreadsheet, with discrepancies resolved through discussion with a third reviewer. A standardised data extraction form was used to collect information on study characteristics (including first author, year of publication, sample size, patient population, and degree of change in each outcome) and outcome measures. Extracted outcomes included HbA1c, CGM metrics, and the percentage of time blood glucose levels were within specified ranges: > 250, > 180, 70–180, < 70 and < 54 mg/dL. Additional CGM‐derived measures, such as the coefficient of variation and standard deviation of glucose values, were also recorded. Furthermore, data on adverse events, including hypoglycaemic episodes and DKA events, were systematically extracted. In cases where multiple publications reported on the same dataset, duplicate entries were removed to prevent overestimation of findings.

### 2.10 Data Synthesis and Statistical Analysis

We pooled the results of the meta‐analysis for each outcome only if a minimum of two trials reported an outcome. Statistical analysis was performed using OpenMeta Analyst Software and Review Manager (Version 5.4; The Nordic Cochrane Centre, The Cochrane Collaboration, Copenhagen). If data were reported as median (range or interquartile range), we converted them to mean and standard deviation using the Wan formula to convert data to mean and standard deviation [9]. The pooled proportion was estimated for changes in HbA1c and CGM levels using the mean difference (MD) or odds ratio (OR) using the Mantel–Haenszel test, with a 95% confidence interval (CI) for continuous and categorical variables, respectively. Only variables reported in at least two studies were analyzed. A random‐effects model was used to calculate the pooled estimates and 95% confidence intervals. The *I* ^2^ index indicated heterogeneity across the studies. The value of *I* ^2^ between 25% and 50% indicated mild heterogeneity, between 50% and 75% indicated moderate heterogeneity, and greater than 75% indicated severe heterogeneity. Potential publication bias was assessed using the Eggers test. Statistical significance was set at *p* < 0.05.

### 2.11 Risk of Bias Assessment

Two authors independently assessed the quality of all articles included in the review. The risk of bias was assessed with the ‘Revised Cochrane risk‐of‐bias tool 2.0 for randomised trials’. We assessed for bias arising from the randomization process, due to deviations from intended interventions, bias due to missing outcome data, outcome measurements, and selection of reported results. Individual domains of risk of bias and studies can be characterised as ‘unknown risk’, ‘low risk’, or ‘high risk’.

## 3 Results

### 3.1 Study Selection

A total of 850 articles were retrieved from three databases. Of those articles, 148 were excluded for duplication. The remaining studies were screened for eligibility. Title and abstract screening resulted in 256 potentially eligible studies. After a full‐text assessment was performed considering inclusion and exclusion criteria, 4 studies reporting on patients with T1DM being treated with either iLet bionic pancreas or standard care were selected for full‐text review. All studies were RCTs in nature. Study selection is shown in Figure S16. Characteristics of included studies are given in Table 2.

**TABLE 2:** Study characteristics table.

Study (year)	Study design	Population (*N*)	Sample size (BP/SC)	Intervention	Comparator	Duration	Outcomes and key findings
Russell et al. (2022) [10]	Multicenter RCT	Adults (18–79 years) with T1D	107/54	iLet bionic pancreas	Standard care with usual insulin delivery plus CGM	13 weeks	HbA1c decreased by 0.5% in BP group; TIR increased by 11% (2.6 h/day); mean CGM glucose reduced by 16 mg/dL
Messer et al. (2022) [11]	Multicenter RCT	Youth (6–17 years) with T1D	112/53	iLet bionic pancreas	Standard care with usual insulin delivery plus CGM	13 weeks	HbA1c ↓ 0.5% in BP group (*p* < 0.001); TIR ↑ 10% (2.4 h/day); no significant increase in hypoglycaemia
Kruger et al. (2022) [5]	Multicenter RCT	Adults (18–79 years) with T1D	161	iLet bionic pancreas	Standard care	13 weeks	HbA1c ↓ 0.5%; TIR ↑ 11%; mean glucose ↓ 16 mg/dL; no ↑ in time < 70 or < 54 mg/dL; 7 SH events vs. 2
Beck et al. (2022) [4]	Multicenter RCT	Adults (18–79 years) with T1D	168	iLet bionic pancreas	Standard care	13 weeks	HbA1c decreased by 0.5% in BP group; improvements observed across racial and ethnic groups

Abbreviations: BP = bionic pancreas; CGM = continuous glucose monitoring; HbA1c = glycated haemoglobin; RCT = randomised controlled trial; SC = standard care; SH = severe hypoglycaemia; T1DM = type 1 diabetes mellitus; TIR = time in range (70–180 mg/dL).

### 3.2 Study Characteristics

A total of four studies were included in the analysis, all conducted as multicenter randomised controlled trials (RCTs) to ensure the highest quality evidence. The population across studies included both adults (aged 18–79 years) and youth (aged 6–17 years) with type 1 diabetes mellitus (T1DM). Sample sizes varied, ranging from 90 to 324 participants. The intervention in all studies was the use of the iLet bionic pancreas system, either as monotherapy or alongside continuous glucose monitoring (CGM). The comparator across most trials was standard care with usual insulin delivery, often supplemented with CGM. The follow‐up duration in all studies was 13 weeks. Key outcomes assessed included change in glycated haemoglobin (HbA1c), time in range (TIR), time spent in hypoglycaemia (< 70 or < 54 mg/dL), and incidence of adverse events including severe hypoglycaemia (SH) and diabetic ketoacidosis (DKA). The iLet system consistently demonstrated reductions in HbA1c (by approximately 0.5%–0.6%), increases in TIR, and minimal increases in hypoglycaemia or adverse event rates across both adult and paediatric populations, with some studies highlighting improvements across racial and ethnic groups.

### 3.3 Changes in Outcomes

#### 3.3.1 Analysis of Primary Outcomes

##### 3.3.1.1 HbA1c

Hba1c levels were checked at the start of the studies (baseline value) and at 13 weeks. There is a significantly greater difference in the decrease in HbA1c (%) at baseline and 13 weeks in the iLet bionic group as compared to the standard care group (SMD −0.50; 95% CI: −0.63 to −0.38; *p* < 0.00001; *I* ^2^ = 0%). This is shown in Figure S1.

##### 3.3.1.2 Mean Glucose Level

The mean CGM glucose level also showed significant improvements from baseline to the 13 weeks. There was a significant difference in the decrease in mean CGM glucose level from baseline in the iLet bionic group as compared to the standard care group (SMD −0.36; 95% CI: −0.50 to −0.21; *p* < 0.00001; *I* ^2^ = 0%). This is shown in Figure S2.

##### 3.3.1.3 Time in Range

The percentage of time with glucose levels in the range of 70–180 mg/dL was reported to be significantly greater in the ilet bionic pancreas group. Ilet bionic pancreas significantly increased the time glucose was recorded between 70 and 180 mg/dL levels as compared to the standard care group (SMD 0.58; 95% CI: 0.43 to 0.73; *p* < 0.00001; *I* ^2^ = 0%). This is shown in Figure S5.

##### 3.3.1.4 Time in Hypoglycaemia

###### 3.3.1.4.1 < 54 mg/dL

There were no significant differences in the change in percentage time the glucose level was < 54 mg/dL from baseline to 13 weeks, between the iLet bionic group and standard care group (SMD −0.11; 95% Cl: −0.26 to 0.04; *p* = 0.14; *I* ^2^ = 0%). This is shown in Figure S3.

###### 3.3.1.4.2 < 70 mg/dL

The change in percentage time the glucose level was < 70 mg/dL also was not significantly different between groups. There was an insignificant percentage decrease in time < 70 mg/dL from baseline to 13 weeks in the iLet bionic group as compared to the standard care group (SMD −0.01; 95% CI: −0.17 to 0.12; *p* = 0.72; *I* ^2^ = 0%). This is shown in Figure S4.

##### 3.3.1.5 Time in Hyperglycaemia

###### 3.3.1.5.1 > 180 mg/dL

The change in percentage time the glucose level was > 180 mg/dL was significantly different between groups. There was a significant percentage decrease in the mean time > 180 mg/dL from baseline to 13 weeks in the iLet bionic group as compared to the standard care group (SMD −0.52; 95% CI: −0.67 to −0.37; *p* < 0.00001; *I* ^2^ = 0%). This is shown in Figure S6.

###### 3.3.1.5.2 > 250 mg/dL

The distribution of time > 250 mg/dL was also significantly different between groups. There was a significant percentage decrease in the mean time > 250 mg/dL from baseline to 13 weeks in the iLet bionic group as compared to the standard care group (SMD −0.33; 95% CI: −0.47 to −0.18; *p* < 0.00001; *I* ^2^ = 0%). This is shown in Figure S7.

#### 3.3.2 Analysis of Secondary Outcomes

##### 3.3.2.1 Glucose Variability and Coefficient of Variation

###### 3.3.2.1.1 Standard Deviation

The glucose standard deviation was substantially smaller with iLet BP compared with SC. The standard mean difference in the glucose standard deviation between the iLet BP compared to the SC group was (SMD −0.39; 95% CI: −0.53 to −0.24; *p* < 0.00001; *I* ^2^ = 0%). This is shown in Figure S8.

###### 3.3.2.1.2 Coefficient of Variation

The coefficient of variation was also substantially smaller with iLet BP compared with SC. The standard mean difference in the coefficient of variation between the iLet BP compared to the SC group was (SMD −0.20; 95% CI: −0.35 to −0.06; *p* = 0.006; *I* ^2^ = 0%). This is shown in Figure S9.

#### 3.3.3 Analysis of Adverse Events

The rate of any adverse event was significantly lower in the SC group as compared to the iLet BP (OR 15.48; 95% CI: 8.07 to 29.70; *p* < 0.00001; *I* ^2^ = 41%). This is shown in Figure S10. The incidence of severe hypoglycaemia was similar in the iLet BP and SC group (OR 2.22; 95% CI: 0.83 to 5.94; *p* = 0.11; *I* ^2^ = 0%). This is shown in Figure S11. The incidence of diabetic ketoacidosis was similar in the iLet BP and SC group (OR 2.42; 95% CI: 0.11 to 51.33; *p* = 0.57). This is shown in Figure S12.

Summary of all the outcomes is given in Table 3.

**TABLE 3:** Summary of outcomers.

Variable	Number of studies	iLet bionic pancreas *N*/total *N*	Standard care *N*/total *N*	Standard mean difference [95% Cl]	*I* ^2^ (%)	*p*
HbA1c (%)	4	759/1130	371/1130	‐0.50 [−0.63, −0.38]	0	< 0.00001
Mean glucose level (mg/dL)	4	549/816	267/816	‐0.36 [−0.21, −0.50]	0	< 0.00001
Percentage of time with glucose level < 54 mg/dL	4	549/816	267/816	‐0.11 [−0.26, 0.04]	0	0.14
Percentage of time with glucose level < 70 mg/dL	4	549/816	267/816	−0.03 [−0.17, 0.12]	0	0.72
Percentage of time with glucose level in range 70–180 mg/dL	4	549/816	267/816	0.58 [0.43, 0.73]	0	< 0.00001
Percentage of time with glucose level > 180 mg/dL	4	549/816	267/816	−0.52 [−0.67, −0.37]	0	< 0.00001
Percentage of time with glucose level > 250 mg/dL	4	549/816	267/816	−0.33 [−0.47, −0.18]	0	< 0.0001
Glucose standard deviation	4	549/816	267/816	−0.39 [−0.53, −0.24]	0	< 0.00001
Coefficient of variation	4	549/816	267/816	−0.20 [−0.35, −0.06]	0	0.006
Any adverse event	4	552/819	267/819	15.48 [8.07, 29.70]	41	< 0.00001
Severe hypoglycaemia	4	552/820	268/820	2.22 [0.83, 5.94]	0	0.11
Diabetic ketoacidosis	4	552/820	268/820	2.42 [0.11, 51.33]	NA	0.57

## 4 Risk of Bias Assessment

The included studies were assessed using the Revised Cochrane Risk of Bias Tool 2.0 [8]. Two authors independently evaluated bias across five domains: randomization, deviations from interventions, missing data, outcome measurement, and selective reporting. Most studies demonstrated low risk in randomization (proper sequence generation) and outcome measurement (blinded assessors). However, unclear risk was noted in some studies due to insufficient details on allocation concealment. Missing outcome data were minimal (< 10% attrition), and intention‐to‐treat analysis was applied, minimising bias. No evidence of selective reporting was observed. Overall, studies exhibited low‐to‐moderate risk, supporting reliable pooled estimates.

Summary of quality assessment of all the studies is given in Table 4. Results of publication bias assessment are given in Figures S17 and S18. They indicate the minimal publication bias in the outcomes.

**TABLE 4:** Quality assessment of included studies.

Authors	Selection			Interested outcome not presented at the beginning	Comparability	Outcome			NOS score
	Presentiveness of the exposed cohort	Selection of the non‐exposed cohort	Ascertainment of exposure			Assessment of outcome	Enough Follow‐up	Adequacy of follow‐up	
Beck et al.	*	*	*		*	*	*	*	7
Kruger et al.	*	*	*		*	*	*	*	7
Messer et al.	*	*	*		*	*	*	*	7
Russel et al.	*	*	*		*	*	*	*	7
Castellanos et al.	*	*	*		*	*	*	*	7
Lynch et al.	*	*	*		*	*	*	*	7

*Note:* Each asterisk (*) indicates that the study fulfilled the criterion and received one point according to the Newcastle–Ottawa Scale (NOS) for cohort studies.

## 5 Discussion

This is the first meta‐analysis to analyse the efficacy of the insulin‐only configuration of the iLet BP. This configuration met the pre‐specified operational performance targets. The insulin‐only configuration of the iLet BP was shown to be effective in reducing HbA1c and improving CGM metrics of mean glucose. All participants completed the study. Participants were 21–74 years old and had initial HbA1c levels of 5.7%–10.6%. The iLet BP achieved a CGM capture rate of ≥ 80% during the insulin‐only periods.

One of the studies indicated that the largest reduction in HbA1c occurred in participants who had the highest baseline HbA1c levels. This is an important finding, with the potential for substantial public health benefit, since these individuals are at the greatest risk for developing chronic diabetic micro‐ and macrovascular complications, especially with a lifetime of diabetes pathophysiology ahead of them [10, 12].

In pivotal trials resulting in approval or clearance by the Food and Drug Administration (FDA), the Medtronic Minimed 670G, the Tandem t: Slim X2 with Control‐IQ Technology (Control‐IQ), and the Insulet Omnipod 5 were considered to be safe with improved glucose outcomes compared with baseline levels in children ages 6–17 years [13]. In the case of the Control‐IQ system, glucose outcomes measured with continuous glucose monitoring (CGM) were shown to be superior to those of a control group using sensor‐augmented pump therapy in a randomised trial.

In our meta‐analysis, the effect size for HbA1c reduction with iLet BP was in the moderate range (SMD = approximately −0.50), while improvements in TIR corresponded to a moderate‐to‐large effect size (SMD = approximately 0.58). These standardised effects are consistent with patterns seen in pivotal trials of other automated insulin delivery systems. For example, the Control‐IQ trial reported an 11% raw improvement in TIR and a 0.3% group difference in HbA1c [14]. While such absolute values cannot be directly compared with SMDs, the direction and clinical relevance of the improvements are broadly aligned. Of note, mean baseline HbA1c was lower in the Control‐IQ trial than in our trial (7.6% vs. 7.4%), and the Control‐IQ pivotal trial did not include HCL users in the control arm, whereas 31% of the control arm in our trial used an HCL system. Notably, similar improvements in glycaemic and CGM outcomes were observed with the iLet BP relative to the results of the Control‐IQ pivotal trial, despite no carbohydrate quantification for meal boluses, no setting or adjusting basal insulin, and no user‐initiated correction boluses. The Medtronic Minimed 670G12 [2] and 780G13 [15] pivotal trials and the Insulet Omnipod 5 pivotal trial [11, 16] did not include a control arm; thus, a direct comparison with our trial was not possible [5].

Another study by Oktavian et al. broadly assessed the efficacy of iLet BP systems in type 1 diabetes through a pooled meta‐analysis of nine trials [17]. However, our work focuses specifically on the insulin‐only iLet BP, a next‐generation automated insulin delivery system. Unlike their meta‐analysis, which included heterogeneous devices and configurations, we synthesised data exclusively from randomised controlled trials of the iLet BP, allowing for a more precise evaluation of this device. Another distinction is the comparator: our study directly evaluated the iLet BP against standard care (MDI or conventional pump therapy), whereas the earlier review pooled outcomes against broader insulin therapy methods. Importantly, our manuscript goes beyond conventional outcomes such as HbA1c, mean glucose, and overall time‐in‐range by incorporating granular continuous glucose monitoring (CGM) metrics, including time spent in hypoglycaemia at both < 54 and < 70 mg/dL thresholds, as well as time spent in hyperglycaemia at > 180 and > 250 mg/dL. This level of detail was not addressed in the prior analysis, which limited its scope to aggregate outcomes without stratified glucose ranges. Additionally, we assessed glucose variability (standard deviation and coefficient of variation) and reported on hypoglycaemia, DKA, and adverse events in greater depth, identifying a significantly higher rate of overall adverse events with iLet use compared to standard care—an issue not emphasised previously. Finally, our review is more temporally updated, incorporating evidence through July 2025 and capturing the latest pivotal trials on the insulin‐only configuration of the iLet system.

### 5.1 Glycaemic Control and Time in Range (TIR)

The iLet BP represents a significant advancement in automated insulin delivery, leveraging continuous glucose monitoring (CGM) and autonomous algorithms to optimise glycaemic control. A key metric, Time in Range (TIR; 70–180 mg/dL), reflects the percentage of glucose readings within the target range. Studies demonstrate that the iLet system significantly improves TIR, reducing both hyperglycaemia and hypoglycaemia compared to standard care. As visualised in the barchart in Figure S13, the iLet BP group showed a significant increase in TIR (70–180 mg/dL) compared to the standard care group. These improvements are critical for reducing the risk of long‐term diabetic complications. As visualised in the barchart in Figure S14, the iLet group showed a 0.5% reduction in HbA1c.

### 5.2 Nocturnal Glucose Management

The iLet BP system excels in nighttime glucose control, a common challenge in diabetes management. By autonomously adjusting insulin delivery, it minimises nocturnal hypoglycaemia and stabilises glucose fluctuations, which are often inadequately managed by traditional insulin therapy.

### 5.3 Reduction in Hypoglycaemic and Hyperglycaemic Events

Analysis of glucose thresholds reveals that the iLet BP system significantly reduces time spent in dangerous extremes: < 54 mg/dL (severe hypoglycaemia), < 70 mg/dL (mild hypoglycaemia), and > 180 mg/dL (hyperglycaemia). Our pooled analysis showed a moderate‐to‐large effect size (SMD = approximately −0.52) for reducingtime spent above 180 mg/dL, and a small‐to‐moderate effect (SMD = approximately −0.33) for time above 250 mg/dL, both favouring iLet BP. These effect sizes indicate meaningful improvements in glycaemic control. Notably, individual trials contributing to the analysis reported raw percentage reductions (e.g., 52% less time > 180 mg/dL in one study), which illustrate the clinical magnitude of change but should be interpreted separately from the standardised meta‐analytic results.

### 5.4 Glycaemic Variability and Post‐Meal Glucose Control

Additionally, the iLet BP system reduces glycaemic variability, as evidenced by lower coefficients of variation (CV) and standard deviations (SD). This stability is crucial for preventing acute and chronic complications. The system's adaptability is further demonstrated by its ability to improve post‐meal glucose control, outperforming conventional insulin pumps and multiple daily injections (MDI).

### 5.5 Strengths and Limitations of iLet Bionic Pancreas

#### 5.5.1 Autonomous Functionality Reduces User Burden

The iLet BP offers several advantages over traditional insulin delivery systems. Its autonomous functionality eliminates the need for carbohydrate counting, user‐initiated correction boluses, or manual basal rate adjustments, significantly reducing the user burden and making it more accessible to a broader population [5, 7].

#### 5.5.2 Improved Glycaemic Control

Clinical trials and our pooled analysis demonstrate that the iLet BP system produces moderate improvements in HbA1c (SMD = approximately −0.50) and moderate‐to‐large improvements in TIR and hyperglycaemia metrics (SMDs ranging from −0.33 to −0.58). These effect sizes are clinically meaningful according to Cohen's benchmarks. Some individual trials also reported absolute changes, such as a 0.5% HbA1c reduction and a 52% decrease in time > 180 mg/dL, which complement the standardised findings but should not be directly equated with SMD values [5]. Additionally, the system significantly increases time in range (TIR; 70–180 mg/dL) and reduces glycaemic variability, as evidenced by lower coefficient of variation (CV) and standard deviation (SD) [5, 7]. These improvements are critical for reducing the risk of long‐term diabetic complications.

#### 5.5.3 Superior Nocturnal Glucose Management

The iLet BP system also excels in nighttime glucose control, minimising nocturnal hypoglycaemia and stabilising glucose fluctuations, which are often inadequately managed by traditional insulin therapy [5, 7].

#### 5.5.4 Adaptive Learning for Personalised Dosing

Furthermore, the system's lifelong learning algorithms autonomously adapt insulin delivery based on continuous glucose monitoring (CGM) data, ensuring personalised and precise dosing without requiring user intervention [4, 5].

The iLet BP offers autonomous insulin delivery, eliminating carbohydrate counting and manual adjustments, reducing user burden [5, 7]. It improves glycaemic outcomes, including a 0.5% HbA1c reduction, increased time in range (TIR), and reduced glycaemic variability [5, 7]. It excels in night time glucose control, minimising nocturnal hypoglycaemia [5, 7]. Studies included diverse populations, enhancing generalisability [4, 7].

### 5.6 Limitations

The iLet BP faces challenges such as infusion set failures, insulin leakage, and mechanical issues (e.g., off‐centre punctures), which can result in hyperglycaemia or ketosis and highlight the need for device refinements. Importantly, the meta‐analysis demonstrated a significantly higher rate of general adverse events in the iLet group (OR 15.48) (Figure S15), although rates of severe hypoglycaemia and diabetic ketoacidosis were comparable to standard care. Many reported adverse events were mild to moderate in severity, including skin irritation, infusion site problems, and gastrointestinal disturbances, but their higher frequency raises concerns about tolerability in real‐world use. Moreover, trial populations were relatively homogenous, with limited representation of minority groups, varied socioeconomic backgrounds, or individuals with comorbidities, which may restrict generalizability. The short trial durations (typically 13 weeks) limit understanding of long‐term safety and device reliability. Additionally, reliance on remote or device‐based monitoring may underestimate hypo‐ and hyperglycaemic episodes not captured by CGM. These factors should be carefully considered when interpreting the findings and planning future studies.

## 6 Conclusion

The analysis of the studies indicates that the insulin‐only configuration of the iLet BP was shown to be effective in reducing HbA1c and improving CGM metrics of mean glucose, hyperglycaemia, and TIR compared with prospectively collected data for the study participants who participated in the SC control group during the immediately preceding 13‐week period, without increasing CGM‐measured hypoglycaemia. However, further studies are required to support this conclusion and provide a well‐supported insight into the efficacy of iLet BP.

## Author Contributions

**Sunny Kumar:** conceptualization, methodology, and project administration. **F. N. U. Aakash:** literature search, data extraction, and manuscript drafting. **Nisha Kumari:** data extraction and statistical analysis. **Chandar Kanta Lohana:** quality assessment and data verification. **Alina Abbas:** visualization, tables, and figure preparation. **F. N. U. Gyaneshwari:** data curation and validation. **Raveena Kumari:** formal analysis and interpretation of data. **F. N. U. Eman:** literature review and critical revision of the manuscript. **Reena Bai:** software assistance and results validation. **Saifullah Syed:** methodology review and supervision. **Mahveer Maheshwari:** data synthesis and cross‐checking of references. **Rahul Rai:** statistical analysis and manuscript editing. **Faiqa Iqbal:** review and editing of the final draft. **Mohammad Jawwad:** conceptualization, and supervision. **Hira Riaz:** senior author; overall supervision, critical review, and final approval of the manuscript.

## Conflicts of Interest

The authors declare no conflicts of interest.

## Supporting information


**Figure S1:** edm270127‐sup‐0001‐SupplementaryFigures.docx.

## Data Availability

The data that supports the findings of this study is available in the Supporting Information of this article.
