# Correction to ‘Efficacy and Safety of iLet Bionic Pancreas in Patients With Type 1 Diabetes Mellitus: A Systematic Review and Meta‐Analysis’

**DOI:** 10.1002/edm2.70150

**Published:** 2026-01-18

S. Kumar, F.N.U. Aakash, N. Kumari, et al., “Efficacy and Safety of iLet Bionic Pancreas in Patients With Type 1 Diabetes Mellitus: A Systematic Review and Meta Analysis,” *Endocrinology, Diabetes & Metabolism* 8, no. 6 (2025): e70127, https://doi.org/10.1002/edm2.70127.

The third affiliation was incorrect for co‐authors Nisha Kumari, Chandar Kanta Lohana, F. N. U. Gyaneshwari, F. N. U. Eman, Reena Bai, Mahveer Maheshwari and Rahul Rai in the published article.

It has been corrected from ‘Liaquat National and Medical College, Karachi, Pakistan’ to ‘Liaquat University of Medical and Health Sciences, Jamshoro, Pakistan’.

We apologise for this error.

