# Supplementary material for: Efficacy and Safety of iLet Bionic Pancreas in Patients With Type 1 Diabetes Mellitus: A Systematic Review and Meta Analysis
Source: Endocrinol Diabetes Metab. 2025 Oct 28;8(6):e70127. doi: 10.1002/edm2.70127 (PMC12568523; doi:10.1002/edm2.70127)
Supplement: Supplementary file 1 — Figure S1: edm270127‐sup‐0001‐SupplementaryFigures.docx. [file EDM2-8-e70127-s001.docx]

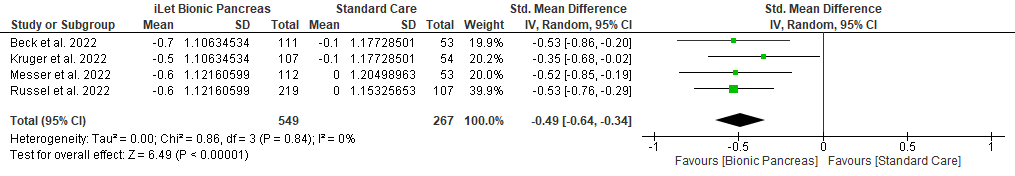


Figure S1. Comparison of HbA1c levels between iLet group and Standard care group.


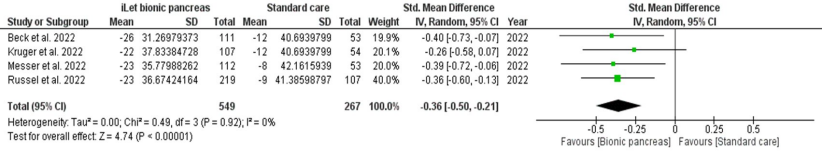
Figure S2. Comparison of reduction in Mean glucose levels between iLet group and Standard care group.
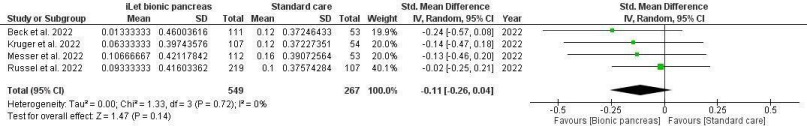


Figure S3. Change in percentage time the glucose level was <54mg/dl between iLet group and Standard care group
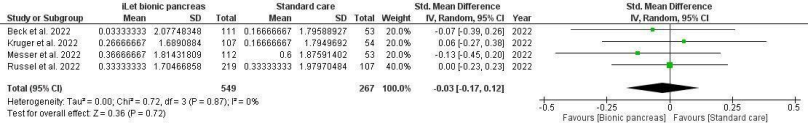
Figure S4. Change in percentage time the glucose level **was** <70mg/d**l** between iLet group and Standard care group.


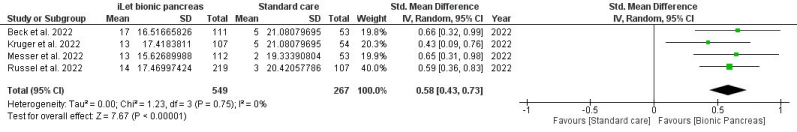
Figure S5. Change in percentage time the glucose level was 70-180mg/dl between iLet group and Standard Care group


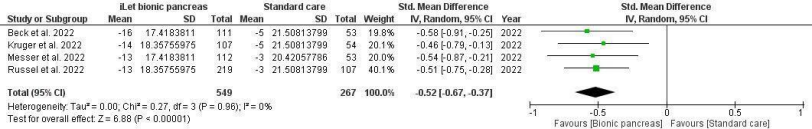
Figure S6. **Change** in percentage time the glucose level was >180mg/dl between iLet group and Standard Care group


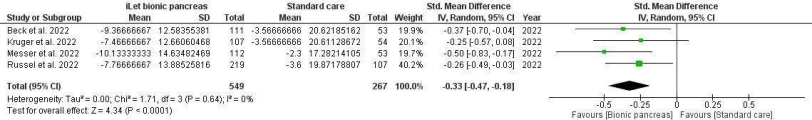
Figure S7. **Change** in percentage time the glucose level was >250mg/dl between iLet group and Standard care group


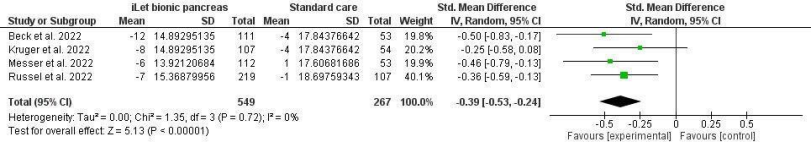
Figure S8. Comparison of Glucose standard deviation between iLet group and Standard care group.


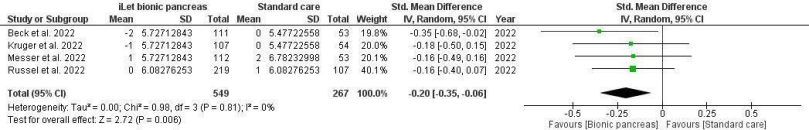
Figure S9. Comparison of **Coefficient** of variation between iLet group and Standard care group.


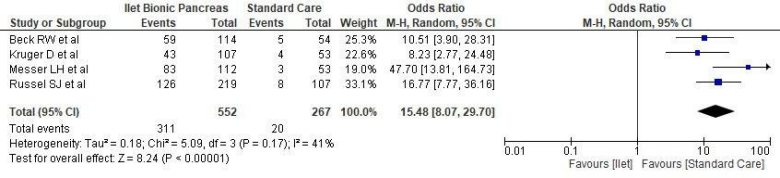
Figure S10. Demonstrating Any adverse event in iLet group and Standard care group


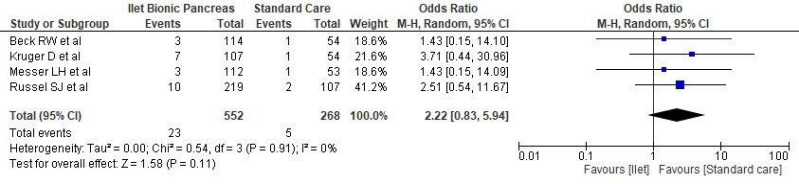
Figure S11. Comparison of Severe Hypoglycemia seen in iLet group and Standard care group.


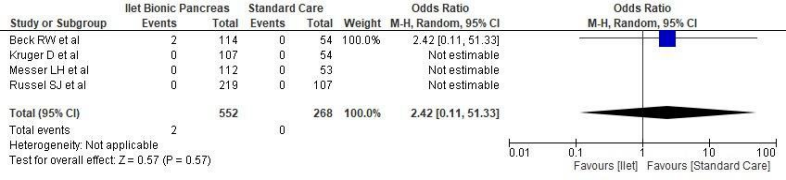
Figure S12. Comparison of Diabetic ketoacidosis seen in iLet group and Standard care group


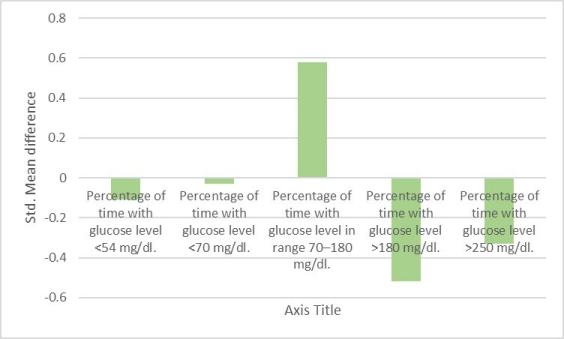


Figure S13. Bar chart showing the SMD for iLet BP and SC for change in time duration the patients had their glucose levels in the mentioned range.


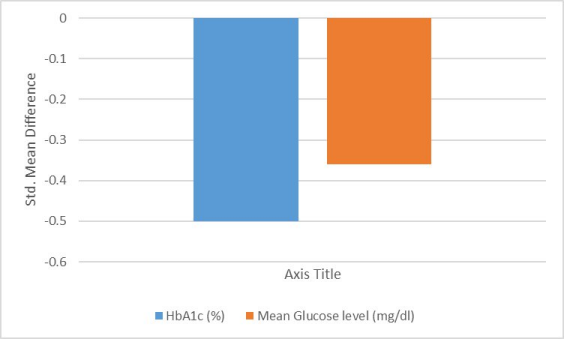
Figure S14. Bar chart showing the SMD for iLet BP and SC for change in HbA1c and Mean glucose levels.


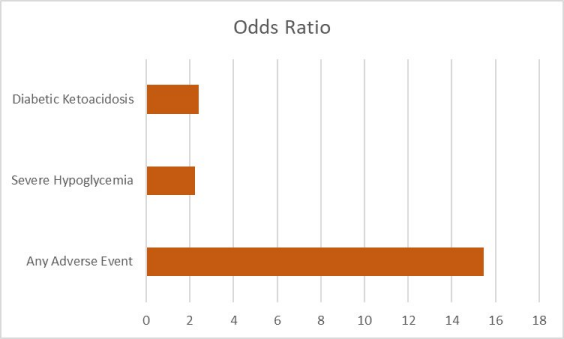


Figure S15. Bar chart showing the Odds Ratios iLet BP and SC for adverse events experienced by the patients after each intervention.


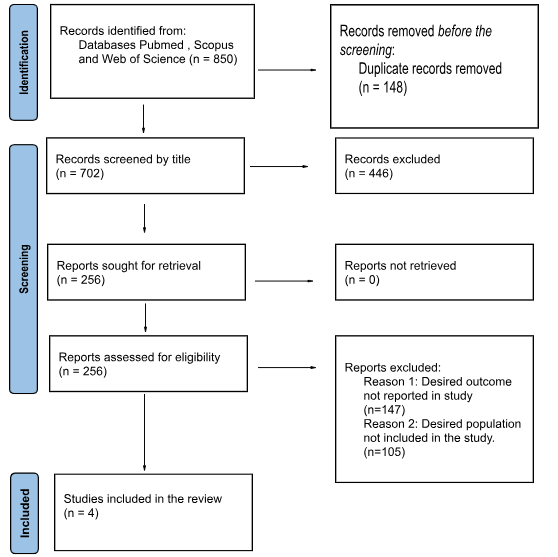


Figure S16. Prisma Flow Chart


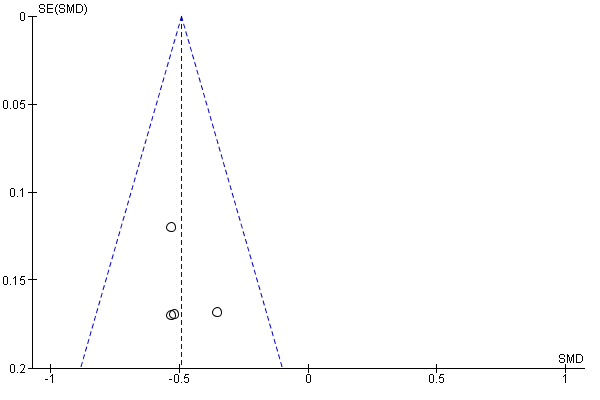


Figure S17. Funnel plot to assess publication bias in mean HbA1c outcome.


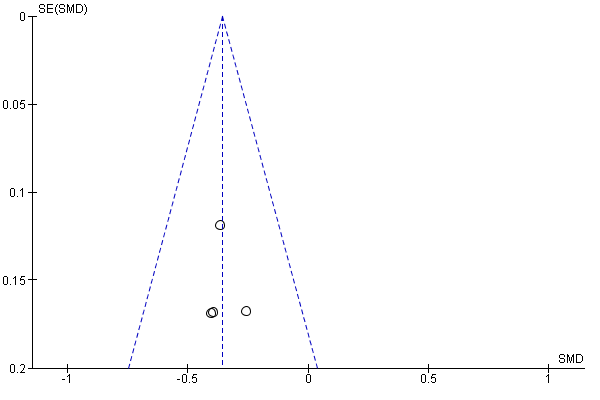


Figure S18. Funnel plot to assess publication bias in Mean glucose level outcome.
